# The utilization of single-cell sequencing technology in investigating the immune microenvironment of ccRCC

**DOI:** 10.3389/fimmu.2023.1276658

**Published:** 2023-11-27

**Authors:** Yuanxin Liu, Guangzhen Wu

**Affiliations:** Department of Urology, The First Affiliated Hospital of Dalian Medical University, Dalian, China

**Keywords:** ScRNA-seq, ccRCC, TME, time, T-cell

## Abstract

The growth and advancement of ccRCC are strongly associated with the presence of immune infiltration and the tumor microenvironment, comprising tumor cells, immune cells, stromal cells, vascular cells, myeloid-derived cells, and extracellular matrix (ECM). Nevertheless, as a result of the diverse and constantly evolving characteristics of the tumor microenvironment, prior advanced sequencing methods have frequently disregarded specific less prevalent cellular traits at varying intervals, thereby concealing their significance. The advancement and widespread use of single-cell sequencing technology enable us to comprehend the source of individual tumor cells and the characteristics of a greater number of individual cells. This, in turn, minimizes the impact of intercellular heterogeneity and temporal heterogeneity of the same cell on experimental outcomes. This review examines the attributes of the tumor microenvironment in ccRCC and provides an overview of the progress made in single-cell sequencing technology and its particular uses in the current focus of immune infiltration in ccRCC.

## 1 Introduction

Studying renal cancer at the transcriptome level is highly important to understand the mechanism of this prevalent tumor in the urinary system and discover effective therapeutic approaches. Over the past few years, numerous research investigations have employed high-through put sequencing to extensively elucidate the transcriptome of kidney cancer tissues (1). Nevertheless, earlier investigations have relied on tissues, which exhibit cellular heterogeneity and typically comprise numerous distinct cell types, often found in varying proportions (2). The signal obtained from conventional second-gen high-throughput gene sequencing represents the mean gene expression level in a cell population, thereby resulting in the omission of information regarding expression variation among individual cells and limiting our ability to actively investigate the tumor microenvironment. In contrast, single-cell sequencing can acquire the genetic data of individual cells, while also isolating and amplifying the genome or transcriptome for analysis using high-throughput sequencing at the individual cell level. With this technology, individual cells’ gene structures and gene expression levels can be revealed, along with their structural variation, copy number variation, and RNA expression levels, enabling scientists to differentiate different types of cells precisely and to study molecular mechanisms at the single-cell level. Understanding human-related diseases and physiological functions greatly benefits from transcriptomics studies at the single-cell level, as they can minimize the structural interference of cellular heterogeneity in tissues. The single cell serves as the fundamental building block of an organism (3), playing a crucial role in its structure and function (4, 5). The inception of single-cell sequencing technology occurred in 2009, with the proposal and publication of the initial single-cell paper by Prof. Fuzhuang Tang from Peking University (6). In recent years, there has been a tremendous increase in the use and popularity of single-cell sequencing. scRNA-seq is a commonly employed sequencing technique that aims to enhance sensitivity, reproducibility, transcript coverage, and cell processing throughput. It serves as a molecular microscope to comprehensively illustrate transcriptome diversity, analyze intercellular heterogeneity, discover novel cell types, and elucidate intercellular relationships (7). The single-cell sequencing procedure begins by separating individual cells from tissues using dissociative enzymes and creating single-cell suspensions. Next, the individual cells are captured and their DNA or RNA is amplified, and gene libraries are constructed due to the limited gene content at the single-cell level. Finally, data analysis is performed. The frequently employed techniques for sequencing individual cells include 1. scRNA-seq (single-cell RNA sequencing) 2. scProteomics-seq (single-cell proteome sequencing) 3. scDNA-seq (single-cell DNA sequencing). Renal cell clear cell carcinoma is extensively infiltrated by different immune system components, but the complete demonstration of the influence of immune heterogeneity on the clinical outcome of ccRCC is still pending. In 2021, Chirag Krishna et al. performed scRNA and TCR sequencing on 167,283 cells obtained from different regions of renal clear cell carcinoma tumors, lymph nodes, healthy kidneys, and peripheral blood. The study included two patients who underwent ICB-resistant therapy and four patients who finished ICB therapy to map the immune landscapes of ccRCC. This examines the present utilization of scFusion-seq and scMethyl-seq in the transcriptomic analysis of renal cell carcinoma using single-cell sequencing technology. In recent years, there has been a growing interest in analyzing the gene expression of renal cell carcinoma using single-cell sequencing technology. One particular focus has been on understanding the tumor microenvironment, particularly renal cell clear cell carcinoma. Additionally, this research aims to provide an overview of the current trends in studying the immune microenvironment of renal clear cell carcinoma. Finally, it summarizes the present state and prospects of immuno-immunotherapy for this type of cancer.

## 2 Renal cell carcinoma

### 2.1 Epidemiology of renal cell carcinoma

Renal cell carcinoma is the sixth most common tumor in men and the tenth most common in women, representing 5% and 3% of all diagnosed tumors, respectively, according to global epidemiological surveys (8). Although the incidence of renal cell carcinoma continues to rise, the detection rate of early-stage renal cell carcinoma and microscopic tumors has simultaneously increased through increased awareness of physical examination and the widespread use of abdominal imaging methods. However, at the time of initial detection, approximately 17% of patients present with tumors that are in advanced stages or have spread to distant parts of the body (9). The World Health Organization reported that in 2018, there were 403,262 fresh instances of kidney cancer detected worldwide (which accounted for 2.2% of all new cancer diagnoses that year). Additionally, there were 175,098 new fatalities attributed to kidney cancer (making up 1.8% of all deaths) (10). The typical age of diagnosis for renal cell carcinoma (RCC) is 64 years, with approximately 85% of kidney tumors being RCC, of which around 70% are clear cell renal carcinoma (ccRCC) (11–13). Factors that impact the 5-year survival rate of renal cancer include tumor stage, the extent of graded tumors locally, metastasis to regional lymph nodes, and the level of tumor metastasis (14–18). Hypertension (19, 20), smoking (21), and obesity (22, 23) are among the factors that increase the risk of developing kidney cancer.

### 2.2 Pathologic staging and genetic features of renal cell carcinoma

In 2022, significant modifications and revisions are present in the fifth release of the WHO Classification of Urological and Male Genital System Tumors. With the ongoing advancement of molecular biology research, there have been fresh revelations regarding the categorization of kidney tumors (24). The fifth edition of the WHO Renal Tumor Classification differs from the fourth edition published in 2016 by providing clearer definitions for seven renal cell carcinomas based on molecular characteristics. It also includes updates to the classification of papillary renal cell carcinomas, eosinophilic solid and cystic renal tumors, and the renaming of clear-cell papillary renal cell carcinoma to clear-cell papillary renal cell tumor. Additionally, the fifth edition introduces the inclusion of eosinophilic solid and cystic renal cell carcinoma (ESC-Rcc) as a morphologically defined type of renal cell carcinoma (24). The fifth edition of the WHO Classification of Tumors has categorized renal cell carcinomas into seven different groups, namely Molecularly defined renal carcinomas, Clear cell renal tumors, Papillary renal tumors, Oncocytic and chromophobe renal tumors, Collecting duct tumors, and Other renal tumors. These seven categories encompass a total of 21 pathological subtypes (25). This article will primarily examine the histologic and molecular features of the three most common subtypes: ccRCC (Clear cell renal cell carcinoma), PRCC (Papillary renal cell carcinoma), and chRCC (Chromophobe renal cell carcinoma). CcRCC is the initial prevalent histological subtype of renal cell carcinoma, representing 70% of the occurrence of renal cell carcinoma (26). Clear cell renal cell carcinoma (ccRCC) is identified histologically by the presence of transparent cytoplasm and groups of honeycomb cells enclosed by the endoplasmic reticulum. Regarding molecular characterization, ccRCC is linked to the absence of the majority or entirety of chromosome 3p (27–29). Over the past few years, there has been a growing amount of research confirming frequent mutations at high frequencies in a range of genes located on chromosome 3p, including Polybromo 1 (PBRM1) (30), SET domain containing 2 (SETD2) (31), and BRCA associated protein 1 (BAP1) (29). Several other genes located on chromosome 3p demonstrate haploinsufficiency, implying that when one allele mutates, the remaining allele can still function normally. However, this only accounts for 50% of the required protein level to sustain regular cellular functions. Notably, the genes MLH1 (32) and SETD2 (33) have been linked to the progression of clear cell renal cell carcinoma. TRACERx studies (34) have shown that chromothripsis is the enabler of chromosome 3p loss, and that fragmented DNA fragments form micronuclei upon acquisition of the nuclear envelope and constitute a corrosive microenvironment that in turn drives DNA damage, mutation, and fragmentation (35). Furthermore, the formation of micronucleus (36) has also been linked to hypoxia, oxidative stress, and various physical alterations in the cell, encompassing pressure, temperature, radiation, UV, and ultrasound.Due to the harsh conditions in renal proximal tubules and renal units, particularly during renal diseases like renal failure, there is a conducive environment for the occurrence of chromosome 3p loss, micronucleus formation, and chromosome fragmentation, leading to the progression of ccRCC (37). Several research studies have indicated that the most prevalent reason for the development of ccRCC, whether in disseminated or familial cases, is the mutation, methylation, or loss of the von Hippel Lindau (VHL) gene (38, 39). The initial cause of ccRCC development is believed to be the alteration or removal of the VHL gene. In familial VHL disease, the loss of the VHL gene in the germline (known as Germline loss) results in autosomal dominant inheritance and triggers the formation of multiple tumors (40). And the overall loss of VHL (somatic loss) often occurs in disseminated ccRCC patients (41, 42). Numerous studies have indicated that HIF2a plays a role in controlling the gene expression of EPO, CCND1, and TGFA. Additionally, HIF2α is a key factor in the development of RCC cancer, crucial for the proliferation of VHL-deficient cells (43). Nonetheless, it has been noted that HIF1α serves as the primary catalyst for RCC carcinogenesis instead of HIF2α (44). This suggests that the significance of these two factors’ roles may vary as the disease progresses through different stages. In 2016, recent research conducted by Désirée Schönenberger and colleagues revealed … experimentally demonstrated that loss of either HIF1α or HIF2α in a Vhl/Trp53-deficient mouse model inhibited tumor formation, suggesting that both are required for tumor initiation in this system (45). Accounting for 15% of renal cell carcinomas (46), papillary renal cell carcinoma (PRCC) is the second most prevalent pathological subtype of renal cell carcinoma. Histologically, this condition is identified by the existence of a fibrovascular center containing tumor cells that are organized in a papillary arrangement (47, 48). Delahunt and Eble initially suggested in 1997 that PRCC can be classified into two subtypes, namely type1, and type2, with type1 being the predominant subtype in PRCC. Histologically, Type 1 is distinguished by a single layer of small cells adorned with minuscule papillae and tubular formations. These structures house cytoplasm with amphiphilic to basophilic properties, along with small, uniform, ovoid nuclei. Type 2 exhibits greater heterogeneity and is distinguished by small papillae containing abundant eosinophilic cytoplasm, along with large, circular nuclei featuring prominent nucleoli. Furthermore, the presence of glomerulonephric papillae, papilledema, foamy macrophages within the papillae, and trachomatous bodies is often observed (49, 50). In 1989, Kovacs suggested that renal tumors be categorized as PRCC when papillary structures make up at least 75% of the tumor (51). The molecular characterization of PRCC involves triploid chromosomes 3q, 7, 8, 12, 16, 17, or 20, along with a deletion of the Y chromosome in male patients (52). Other studies have found chromosomes 7 and 17 to be triploid in PRCC to be the only karyotypic variations found in a variety of tumors (53). The observations strongly indicate that papillary renal carcinoma is distinguished by gene duplications on chromosomes 7 and 17 during early development. This distinction is different from the loss of chromosome 3p in clear cell renal cell carcinoma, leading to the reclassification of PRCC. The 2018 review on PRCC highlighted MET, NF2, SETD2, and Nrf2 as the genes linked to the formation of hereditary PRCC. Using second-generation high-throughput sequencing technology, additional genes like FAT1, BAP1, PBRM1, STAG2, NFE2L2, and TP53 have been discovered to potentially relate to the advancement of PRCC. However, the precise mechanism remains unclear and will not be extensively discussed in this paper (48). ChRCC, also known as renal smectic cell carcinoma, makes up 5% of all cases of renal cell carcinoma, ranking as the third most prevalent subtype (54). Histopathologically, ChRCC arises from intercalated cells located in the collecting ducts (55). ChRCC is a clearly defined, non-enclosed tumor with compact, uniform, and pale yellow to deep brown areas. Visible to the naked eye is bleeding, tissue death, formation of cysts, and scarring at the center (46). Chromosomal mutations in chRCC often materialize as whole or partial deletions of chromosomes 1, 2, 6, 10, 13, and 17, allowing ChRCC to be distinguished from other renal cancers (56). In 2014, Caleb F Davis et al. also found that chromosomes 3, 5, 8, 9, 11, 18, and 2 are also significantly missing in chess. In chess, TP53 and PTEN were identified as the most frequent mutations, whereas MTOR, NRAS, TSC1, and TSC2 mutations were uncommon (57). Furthermore, alterations in the promoter region of the TERT gene, responsible for regulating the telomerase enzyme involved in chromosome elongation, result in elevated TERT expression and the occurrence of kataegis mutations. These mutations could potentially play a significant role in the development of RCC (58).

## 3 Evolution of single-cell sequencing technologies

Next-Generation Sequencing (NGS) is an efficient, cost-effective, and accurate gene sequencing technology, and with its popularity, tremendous progress has been made in population genomes and individual genomes. Nonetheless, the gene’s expression value acquired through this technique represents the average expression level across numerous cells, disregarding cellular heterogeneity. Since characteristics of larger cell populations in tissues are more easily observable, while less common characteristics within those thinly populated cells are often concealed, NGS might not offer an understanding of whether variations in expression between samples are influenced by alterations in cellular composition or by alterations in the underlying phenotype. The fundamental building blocks and operational components of an organism are individual cells (3). Typically, distinct cells assume specific functions by collaborating to fulfill the overall purpose of a tissue or an organ. To gain deeper insights into crucial biological processes, it becomes necessary to isolate individual cells and proceed with sequencing, library construction, and subsequent analysis. In the past, single-cell sequencing involved the laborious task of manually isolating cells, resulting in slow progress and limited capacity. However, in 2011, a breakthrough was made with the introduction of single-cell whole genome sequencing (scWGS), which combined the techniques of amplifying single-cell genomes and using high-through put sequencing technologies. This advancement has opened up remarkable possibilities for groundbreaking discoveries in crucial biological fields (59). There are remarkable chances for novel findings in significant domains of biology (59). In recent years, the field of biological sciences has witnessed a significant rise in the popularity of single-cell sequencing technology due to ongoing advancements in technology. Single-cell sequencing can be classified into various types based on different sequencing methods and analysis strategies. scRNA-seq is the prevailing technique for quantifying gene expression at the single-cell level. The technique has been utilized to uncover novel cell categories (60), investigate the kinetics of developmental biological procedures (61), and determine gene regulatory mechanisms. This paper will focus on summarizing the common types of scRNA-seq currently available and their advantages and disadvantages in this section. The analysis of genomic variation and DNA modification patterns in individual cells is often performed using scDNA-seq (Single-cell DNA sequencing) (62). Studying the heterogeneity of individual cells can be beneficial in understanding human development, tumorigenesis, and clonal expansion. scProteomics, also known as single-cell proteome sequencing, is employed to uncover variations in protein expression levels among distinct cell types at the individual cell level. Studying the DNA of an individual cell can be beneficial. Through the assessment of the methylation condition of individual cells, one can uncover variances in cellular category and condition, along with the significance of methylation in the progression of organisms, ailments, and reactions to medication. Initially, scRNA-seq methods involved the isolation of single cells through methods like limited dilution, micromanipulation (micromanipulation) (63), laser capture microdissection (LCM) (64), and flow cytometry. Subsequently, the cells were subjected to cell lysis, reverse transcription, mRNA extraction, amplification, and other procedures, before constructing sequencing libraries separately. Early scRNA-seq technology was used to isolate individual cells by limited dilution method, Micromanipulation (63), laser capture microdissection (LCM) (64), flow cytometry, etc., and then perform cell lysis, reverse transcription, mRNA extraction, amplification, and other processes, and then construct sequencing libraries independently. Limited by cell isolation techniques and high costs, these single-cell sequencing techniques can only detect a small number of cells (tens to hundreds). However, with the emergence of single-cell identification technologies based on barcode tags and novel single-cell separation technologies based on microdroplets or microtiter wells, such as Drop-Seq, Cyto-Seq, etc., the cost of single-cell isolation and capture sequencing has been greatly reduced, enabling single-cell RNA sequencing to enter the high-throughput era. Of course, transcriptome sequencing methods based on individual cell isolation methods are constantly being updated to achieve the goals of saving time and cost, as well as pursuing high accuracy and sensitivity.

### 3.1 Methods of single-cell sequencing and their advantages and disadvantages

By reviewing the literature, we reviewed more than a dozen single-cell sequencing technologies that have emerged in recent years, summarized them in a smooth chronological order, and attempted to list their advantages and disadvantages.

### 3.2 CEL-seq

The CEL-seq method (65) (Cell expression by linear amplification and sequencing) is an early-appearing single-cell sequencing technology that was developed in 2012. This is achieved by using a primer that contains an anchored poly dT, a distinct barcode marker (barcode), an Illumina sequencing junction at the 5’ end, and a T7 promoter. The resulting cDNAs are then combined to create enough templates for *in vitro* transcription (IVT) reactions. Next, the enhanced RNA underwent focused RNA library construction. The RNA is fragmented to a suitable length for sequencing. Then, an Illumina 3’ sequencing junction is added through ligation. After that, it is reverse-transcribed into DNA. The bipartite sequencing of the fragment’s 3’ end, which includes the Illumina junction and the Barcode, is performed. In this sequencing, the Read1 sequence contains the barcode, while the Read2 contains the mRNA sequence. CEL-Seq is a sequencing technique that utilizes linear amplification. However, it stands out due to its optimized manual steps compared to other linear amplification methods. On the downside, it has a relatively low sequencing throughput since only the 3’ end of the transcript is sequenced.

### 3.3 Smart seq2

Smart seq2 method (66) (Switching Mechanism at 5 End of RNA Template) sequencing technology was first proposed in 2013. The procedure is as follows: individual cells are broken down in a solution that includes unrestricted deoxyribonucleotide triphosphates (dNTPs) and oligo(dT) oligonucleotides with a universal anchoring sequence at the 5’ end. Reverse transcription is performed by introducing Moloney Murine Leukemia Virus (MMLV) reverse transcriptase. Once the synthesis reaches the 5’ terminus of the RNA molecule in the template, the reverse transcriptase’s unique terminal transferase activity is utilized to append three cytosines to the 3’ terminus of the complementary DNA (cDNA). The reverse transcriptase enzyme adds three cytosines to the 3’ terminal of the cDNA. After replacing the template, the TSO (template-switching oligo) primer is used to synthesize the second strand of the cDNA, replacing the RNA that is complementary to the first-strand cDNA. Three guanines at the 3’ end of TSO complement the three cytosines at the 3’ end of the first chain. Ultimately, the DNA was disrupted utilizing an altered Tn5 transposase with increased effectiveness, simultaneously incorporating the junctions to both extremities of the cDNA. Subsequently, double-stranded cDNAs suitable for library sequencing were acquired through PCR amplification (66). The fundamental concept of Smart seq2 is based on the initial utilization of MMLVRT (Moloney murine leukemia virus reverse transcriptase), which possesses not just the ability to perform reverse transcription, but also exhibits template conversion and terminal transferase activity. Additionally, during reverse transcription, it can append a small number of cytosines to the 3’ terminus of the freshly synthesized cDNA end of the newly synthesized cDNA. Secondly, there is the unique template-switching oligonucleotide (TSO) that is used as the target of template-switching during template substitution. In terms of genetics, these factors, along with increased stability, led to improvements in the size and production of cDNA libraries created from individual cells. Additionally, there were enhancements in assay coverage, bias, and accuracy (67).

### 3.4 Quartz-seq2

Quartz-seq2 (68) is a single-cell sequencing method with high accuracy and the highest score in benchmarking tests, developed by RIKEN in 2013. In Quartz-seq, an RT primer removal step is incorporated and a suppression PCR method is employed to minimize the production of unwanted by-products. The particular method involved the screening of individual cells using FACS and subsequently lysing them. Using RT primers that include the PCR target region, the mRNA was transcribed in reverse to form first strand cDNA. Nucleic Acid Exonuclease 1 was used to digest the unreacted RT primers, and poly(A) tails were subsequently added to the cDNA’s 3’ end along with any remaining RT primers. Poly(dT) sequences were used in the synthesis of second-strand cDNA, resulting in the production of cDNA and byproducts that contained both labeled and RT primer sequences, as well as whole transcriptome amplification (WTA) articulator sequences. These DNAs are then subjected to suppression PCR to remove the byproducts and obtain high-quality cDNAs for Illumina sequencing. In addition to evaluating the variability in gene expression among cells of the identical category, quartz-seq2 also identifies variations in gene expression among cells in the identical phase of the cell cycle.

### 3.5 SCRB-seq

SCRB-seq (Single-cell RNA barcoding and sequencing) (69) was proposed in 2014, which allows the analysis of mRNAs from a large number of cells using minimal amounts of reagents and sequencing reads per cell. SCRB, unlike Smart-seq, utilizes unique molecular identifiers (UMI) to primarily enrich for RNA 3’ during cDNA amplification by PCR, similar to Smaet-seq, which analyzes the entire length of the mRNA (69). SCRB is comparable to Smaet-seq as both utilize PCR amplification for amplified cDNAs. However, SCRB distinguishes itself from Smart-seq by incorporating unique molecular identifiers (UMIs) to primarily enhance RNA 3’ enrichment. In contrast, Smart-seq examines the entire mRNA length, which differs from SCRB.

### 3.6 MARS-seq

The MARS-seq method (70) was first proposed and validated in 2014 by Diego Adhemar Jaitin et al. MARS-seq can be used to analyze the *in vivo* transcriptional status of thousands of single cells. To begin, individual cells are sorted using FACS into 384-well plates and subjected to reverse transcription (RT) with T7 promoter, partial Illumina articulators, cell barcodes, UMI, and poly(T) primers. Following this, the merged and labeled material undergoes automated processing with three levels of barcoding (molecular, cellular, and plate level), resulting in a substantial enhancement in throughput and reproducibility. This technique can be utilized to classify cell types and cell states and establish connections between them and comprehensive transcriptome profiles across the entire genome.

### 3.7 SUPeR-seq

The SUPeR-seq method (71), which was first proposed in 2015, is a homopolymer-plus-tailed PCR-based scRNA-seq method for sequencing both polyadenylated and non-RNA polyadenylated RNA. ExoSAP-IT effectively eliminates any remaining unreacted primers. Polyadenylation tails were appended to the 3’ terminal of the initial strand complementary DNA (cDNA) by incorporating dATP containing 1% ddATP. To synthesize the second strand cDNA, various Poly(T) primers with distinct anchoring sequences (AnchorY-T24) were employed. Subsequently, PCR was performed using AnchorY-T24 and AnchorX-T15 primers before deep sequencing. However, SUPeR-seq has limitations such as a time-consuming cell isolation process and low throughput (71). The SUPeR-seq technique utilizes homopolymer-plus-tailed PCR to sequence both polyadenylated and non-polyadenylated RNAs (circRNAs) in scRNA-seq. ExoSAP-IT effectively eliminates any remaining unreacted primers. Polyadenylation tails were appended to the 3’ terminal of the initial strand complementary DNA (cDNA) by incorporating dATP containing 1% ddATP. To synthesize the second strand cDNA, various Poly(T) primers with distinct anchoring sequences (AnchorY-T24) were employed. Subsequently, PCR was performed using AnchorY-T24 and AnchorX-T15 primers before deep sequencing. However, SUPeR-seq has limitations such as a time-consuming cell isolation process and low throughput.

### 3.8 Drop-seq

The Drop-seq technique (72), created by a group of scientists at Harvard University in 2015 and documented in the journal CELL, enables the swift examination of numerous individual cells and represents a significant breakthrough in single-cell transcriptome sequencing. By employing a microfluidic apparatus, it can trap microbeads containing barcodes within minuscule droplets alongside cells, thus creating a rapid, cost-effective, and high-capacity approach for sequencing individual cells. The initial step involves the isolation of individual cells from complex tissues, which are then enclosed in droplets along with microbeads containing barcode primers. The droplets are used to lyse individual cells, resulting in the production of mRNAs that attach to primers located on the microbeads. Following that, the mRNAs undergo reverse transcription to produce cDNAs, resulting in the creation of STAMPs (microparticles attached to single-cell transcriptomes). Afterward, these STAMPs containing barcodes can be utilized for high-throughput sequencing and depend on the barcode to examine the cellular source of individual cell transcripts. PCR amplification of STAMPs is facilitated by a shared sequence found in all primers on Drop-seq microbeads. Additionally, the primer sequences include a 30bp oligo dT sequence at the conclusion to capture transcripts. Every microbead consists of over 108 distinct primers that have a shared barcode and unique molecular identifiers (UMIs). Formally, Drop-seq enables the sequencing of a vast quantity of cells simultaneously and the differentiation of the amplification outcomes of distinct transcripts within each cell. Naturally, Drop-seq also possesses a drawback. Specifically, Drop-seq beads and cells are enclosed within the droplet through Poisson distribution, leading to a reduced count of productive droplets with a low effective cell capture rate. Moreover, within the droplet, only the mRNA-capturing process can be accomplished, followed by a subsequent set of reactions, hereby introducing a potential risk of cellular cross-contamination.

### 3.9 In Drop

The In Drop technology (73) was developed in 2015 and, similar to Drop-seq, involves the separation of cells in tiny droplets containing barcoded primers for amplification. However, they also differ in that the amplification principle of in Drop is based on IVT, similar to CEL-seq. in the droplet system, cells are encapsulated in droplets containing lysis buffer, reverse transcription mixture, and hydrogel microspheres carrying barcode primers. Once the droplet is enclosed, the primers are liberated from the hydrogel microspheres through exposure to UV radiation. The reverse transcription process occurs inside each droplet, where the cDNAs are labeled with the corresponding barcode. Afterward, the tiny particles are fragmented, and the substance from every cell is amplified linearly before undergoing sequencing. In comparison to Drop-seq, the inDrop technique yields approximately 150,000 barcodes, resulting in a lower capacity for processing cells in a single operation. However, it captures a higher proportion of cells compared to Drop-seq because of the enhanced effectiveness of its hydrogel microspheres in sealing within the droplets (reducing the likelihood of droplets without microspheres). Nonetheless, the light-sensitive component utilized in the interop technique is expensive and necessitates the operation of a darkroom to avoid light. This process is technically challenging, and exposure to UV radiation could potentially harm cells.

### 3.10 Microwell-Seq

Microwell-Seq (74) is an inexpensive, high-capacity method for sequencing RNA from single cells that is achieved by modifying Cyto-seq. In this technique, cells are separated into agarose microarrays, and their transcripts are collected using magnetic beads. The basic principle of Microwell-seq is the same as that of Cyto-seq, but it uses photolithography to fabricate microporous matrix silicon wafers (microporosity of 28 um in diameter, 35 um in depth, and 100,000 microporosities), which are used as molds to fabricate PDMS (polydimethylsiloxane) chips. Silicon and PDMS chips, which can be reused, are utilized for producing numerous agarose microarrays. Generally, each microwell can accommodate only a single cell, and a solitary plate can simultaneously capture approximately 10,000 individual cells. Although Microwell-Seq has enhanced the complete depiction of cell segregation and offers the advantage of cost-effectiveness with agarose microtiter plates, the issue of cell-to-cell cross-contamination remains a significant challenge encountered in microtiter plate techniques.

### 3.11 Cyto-seq

Cyto-seq was developed in 2015 by H Christina Fan et al. It is also a high-throughput scRNA-seq sequencing method by uses microtiter plates for cell isolation. To a microtiter plate with 100,000 wells, a suspension of cells is introduced and the concentration is regulated to approximately 1 cell per 10 wells. Subsequently, magnetic beads are included in the plate, occupying the majority of the wells. Probes enriched on magnetic beads capture transcripts in the cells, which consist of a universal PCR priming site, a cellular marker, a molecular label, and a ploy tail for mRNA capture. Following the incubation of the beads and cells in a lysis solution, the beads containing captured transcripts were recovered using a magnet. Afterward, the retrieved substances were transcribed in reverse, amplified to create a library, and uniformly sequenced. Cyto-seq possesses a magnetic bead in the majority of the microtiter wells, thus resulting in a slightly higher rate of cell capture compared to Drop-seq. However, it solely accomplishes the mRNA capture process within the microtiter wells. The subsequent collection of all the beads and a series of subsequent reactions increases the likelihood of cross-contamination between cells.

### 3.12 MATQ-seq

The MATQ-seq approach is appropriate for identifying transcriptomes in diverse populations. It draws inspiration from the MALBAC technique, which involves multiple annealing and looping-based amplification cycles. The method is inspired by the primers used in the MALBAC technique and uses primers consisting mainly of G, A, and T bases. Simultaneously, to mitigate the bias caused by PCR amplification after the synthesis of the second strand, this technique employs random hexamers’ distinct molecular identifiers to tag the molecules before amplification and sequencing. MATQ-seq demonstrated greater sensitivity compared to Smart-seq 2 and SUPeR-seq. Furthermore, MATQ-seq exhibited a higher capability in detecting genes with low abundance in comparison to Smart-seq 2. In general, MATQ-seq offered excellent precision and responsiveness in identifying variations in the transcriptome among individual cells of comparable cell types. Nonetheless, the drawback of MATQ-seq involves employing a mouth pipette to separate cells, resulting in a time-consuming and low throughput process (75).

### 3.13 10xGenomics

The Drop-Seq technology, which was enhanced and brought into the market, gave rise to 10xGenomics. As part of the platform’s single-cell RNA-Seq technology, the GemCode technology is used to mix Gel Beads with barcodes, UMIs (Unique Molecular Index), primers, and enzymes by controlling the entry of microfluidic fluid, enabling large-scale single-cell isolation and library construction using the droplet method. To achieve high-throughput isolation of individual cells and construction of single-cell libraries, the Molecular Index, primers, and enzymes containing barcodes and UMIs are combined with single cells. The droplet encapsulation of the 10X Genomics platform has approximately 50% capture efficiency and can be completed in less than 6 minutes. Following the separation process, a structure of GEM (Gel in Emulsion) droplets is created, encompassing a gel bead, a collection of necessary reagents for the reaction, and the target cells. A distinct and organized identification is assigned to every droplet using a collection of 750,000 barcode sequences. These sequences are then analyzed online and split using an information analysis technique. This enables the isolation of 100-10000 cells and the construction of a library simultaneously. 10xGenomics, like In-Drop, utilizes gel beads to introduce oligonucleotides, with both lysis and reverse transcription occurring within the droplet. It greatly simplifies the overall cell lysis to PCR processing time (<10 hours). By employing this method, the implementation of 10x barcodes greatly enhances throughput in comparison to the currently used droplet-based techniques. By utilizing this feature, thousands of cells can be processed in parallel for scRNA-seq (67).

**Figure 1:**
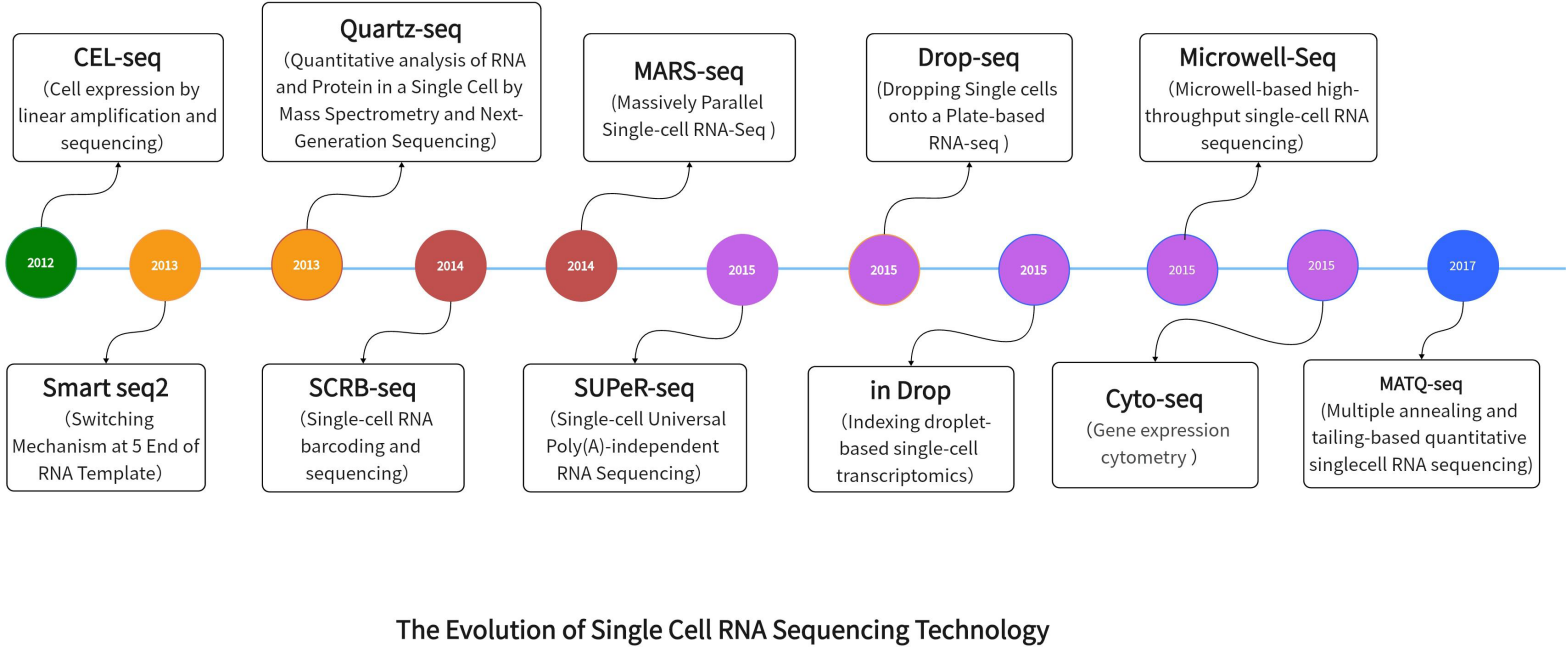
The Evolution of Single Cell RNA Sequencing Technology.

To summarize, we will provide an overview of the progress made in single-cell technology (**Figure 1**). Subsequently, our main emphasis will be on exploring the utilization of single-cell technology in the tumor microenvironment, an area that has gained significant attention in recent times.

## 4 Application of single-cell sequencing technology to the study of the tumor microenvironment in renal cell carcinoma

**Figure 2:**
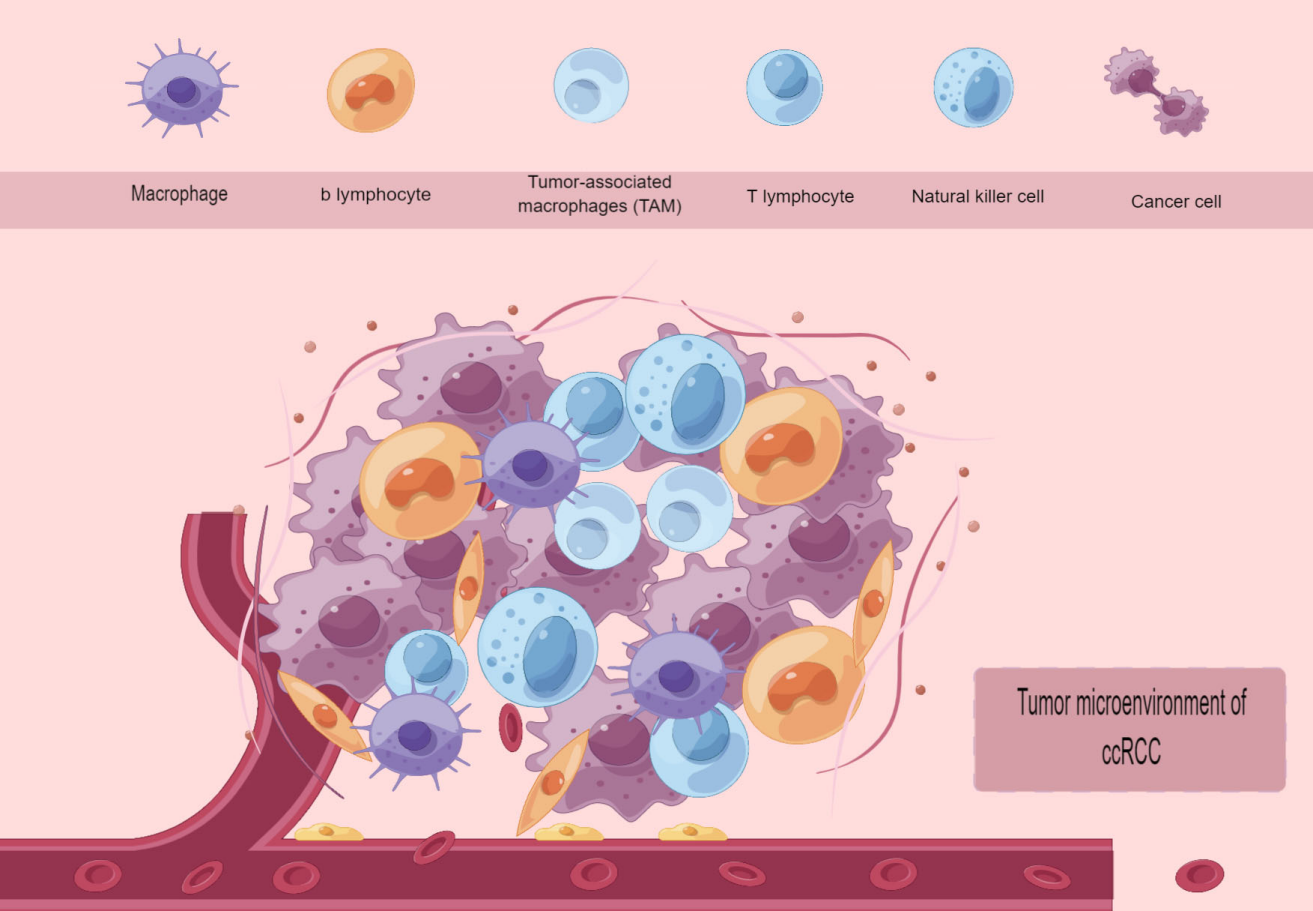
Tumor microenvironment of ccRCC.

The progression of tumors heavily relies on the tumor microenvironment (TME), which is composed of an intricate network of various stromal cells (such as fibroblasts, lymphocytes, macrophages, and endothelial cells) as well as immune cells (including T and B lymphocytes, among others). This includes intracellular substances (such as proteins, enzymes, and nucleic acids), as well as substances found outside the cell (like cytokines, growth factors, hormones, and the extracellular matrix ECM). The tumor cells are surrounded by and receive nourishment from the vascular system (**Figure 2**). The tumor microenvironment is typically divided into two groups: inflammatory TEM and non-inflammatory TEM. Inflammatory TEM is composed of immune cells and pro-inflammatory cytokines, creating an environment that stimulates the immune system and encourages tumor rejection. Non-inflammatory TEM is distinguished by the lack of immune cells and the existence of immunosuppressive elements that create an environment suppressing the immune system, thereby facilitating tumor development (76, 77). With the widespread use of high-throughput RNA sequencing technology, numerous studies have validated that certain significant genetic changes contribute to the development of ccRCC. These alterations include frequent loss of both alleles of tumor suppressor genes on chromosome 3p, such as VHL (90%), PBRM1, SETD2, and BAP1 in ccRCC (78), as well as recurrent loss of one allele of chromosomes 1, 2, 6, 10, 13, and 17, and TP53 mutation, which are noteworthy genetic changes in chRCC (57). Typically, we assume that various RCC subtypes are derived from distinct types of tubular epithelial cells within the renal unit (79). In other words, the potential cell of origin for RCC (referred to as P-CO) can be determined by identifying similar transcriptional patterns between the tumor epithelium of a specific renal cancer subtype and normal renal tissues. Hence, determining the transcriptional profile of P-CO will aid in comprehending the gene expression pattern of tumor epithelial cells. The acquisition of this knowledge will assist in improving disease models in living organisms and promoting the investigation of connections between physical traits and genetic characteristics for different types of diseases (80). In highly vascularized ccRCC subtypes (78), there is a significant presence of immune cells, making antiangiogenic drug therapy and immunotherapy highly effective in advanced ccRCC (81, 82). In chRCC, a type of kidney cancer, as well as in approximately 50% of PRCC, the presence of immune cells is lacking, resulting in reduced effectiveness of immunotherapy and antiangiogenic medications. As previously stated, the transcriptional characteristics of noncancerous kidney tissues and different types of RCC acquired through second-generation high-throughput RNA sequencing (RNA-seq) solely present a mean gene expression profile of all cellular categories in each tissue, disregarding the diversity between cells. In the year 2021, Yuping Zhang and colleagues utilized single-cell RNA sequencing to create cellular diagrams of noncancerous kidney tissues as well as two prevalent forms of RCC (ccRCC/chRCC). They also made predictions about the potential source cell for over 10 subcategories of RCC. The revelation of the potential involvement of renal tumor epithelial cells in promoting immune cell infiltration and other molecular characteristics in the tumor microenvironment was achieved by directing attention toward cell subtypes in various compartments (80). In 2022, Yue Shi and colleagues conducted a study where they utilized single-cell RNA sequencing to analyze 19 surgical tissue samples from 8 individuals diagnosed with clear cell renal cell carcinoma (ccRCC) accompanied by a cancer thrombus. The findings revealed that the tumor thrombus led to an augmentation in the quantity of tissue-resident CD8+ T cells exhibiting characteristics of progenitor cell depletion, in comparison to the corresponding primary tumor. Significantly, TTs (Venous tumor thrombus) displayed augmented extracellular matrix remodeling, involving macrophages, cancerous cells, endothelial cells, and myofibroblasts (83). The utilization of single-cell RNA sequencing technology enables the identification of tumor subtypes through the creation of cellular maps, which facilitates the analysis of the cellular source of the comparative tumors. Additionally, this technology aids in uncovering the functions of diverse immune cells during the progression of tumor growth. Through literature review, it has been discovered that the present utilization of single-cell sequencing technology for examining the immune microenvironment tumor immune microenvironment (TIME) of ccRCC aims to derive additional findings. Consequently, this article will concentrate on summarizing the implementation of single-cell sequencing in ccRCC research and forecasting future advancements in immunotherapy.

### 4.1 Application of single-cell sequencing technology in the exploration of the ccRCC tumor immune microenvironment

Tumors can hinder the immune system’s eradication of tumor cells by modifying how antigens are presented, activating immune checkpoint pathways, and attracting immunosuppressive cells, consequently establishing an environment that suppresses the immune response (84). Tumor cells in ccRCC can downregulate the expression of major histocompatibility complex molecules (MHC), leading to the attenuation of antigen presentation to T cells. This immune escape phenomenon occurs due to the reduced MHC expression, resulting in the absence of MHC molecules in ccRCC. Furthermore, tumor cells can up-regulate the expression of immune checkpoint molecules that hinder T cell activation, including programmed cell death protein 1 (PD-1) and cytotoxic T-lymphocyte-associated protein 4 (CTLA-4), as depicted in (85). In the tumor microenvironment, tumor cells can attract different types of cells that induce immunosuppression. These cells include MDSCs, TAMs, and treg, and they can hinder the function of T cells while facilitating the growth and spread of the tumor. As mentioned previously, high infiltration of TAMs will lead to a poorer prognosis. On the other hand,Treg can suppress T cell activation and facilitate evasion of the tumor’s immune system by generating TGFb and IL-10 (86). The above mechanisms constitute the microenvironment of tumor immunosuppression. The analysis of individual cells in ccRCC offers a broader and more intricate comprehension of the immune microenvironment within the tumor, presenting a fresh method to identify molecular biomarkers derived from immune cells that infiltrate the tumor. Like numerous other forms of cancer, ccRCC is linked to intricate TIME, making it crucial to comprehend the connections between the tumor and the immune system for the advancement of novel therapeutic approaches. In ccRCC, TIME is characterized by the existence of infiltrating immune cells, such as T cells, B cells, natural killer (NK) cells, dendritic cells (DCs), and myeloid-derived suppressor cells (MDSCs) (87). ccRCC exhibits an intricate and ever-changing TIME, encompassing both elements that enhance the immune response and elements that hinder it (88). In ccRCC, CD 8+ T cells exhibit a greater proportion of CD 8+ T cells compared to regulatory T cells (Treg), among other cell types. Furthermore, the presence of tumor-infiltrating lymphocytes (TIL) is linked to a positive outlook in ccRCC. ccRCC additionally exhibits a substantial concentration of MDSC, which possesses the ability to impede immune responses against tumors (89). Furthermore, ccRCC can enhance the expression of immune checkpoint molecules, like programmed death ligand I (PD-L1), which hinders T-cell activation and facilitates tumor immune evasion (90).

#### 4.1.1 T-cell

In various tumors, including ccRCC, CD8(+) T cells play a crucial role in influencing the immune response against tumors, and the prognosis of tumor patients is frequently associated with the level of CD8(+) T cell infiltration (91). While Treg and TAM have been linked to pro-tumorigenic roles, CD8 (+) T cells have been associated with better clinical prognosis and response to immunotherapy (92–94). The effectiveness of immune checkpoint blockade therapy (ICB) may be attributed to the anti-tumorigenic function of CD8 (+) T cells targeting specific antigens (95, 96). It is important to note that the use of antibodies to inhibit the interaction between PD1 and its ligand PD-L1 in immune checkpoint blockade therapy (ICB) has been highly effective in treating cancer. However, the specific mechanism remains unknown because quantitative data on the distribution of clonal expansion of T cells in individual cancer patients is currently unavailable. WU et al. in the year 2020 Examined the characteristics of different T-cell and T-cell receptor groups in neighboring healthy tissues and peripheral blood using advanced single-cell sequencing of RNAs and T-cell receptors from individuals with diverse cancer types. This revealed expansions of effector-like T-cells with similar genetic makeup not just within tumors, but also in neighboring healthy tissues. The most favorable response to anti-PD-L1 therapy (97) was observed in patients exhibiting this genetic profile characterized by clonal-type expansion. David A et al. published their findings in the same year. Conducted a transcriptomic analysis on individual immune cells in tumors and neighboring non-tumor tissues of 13 ccRCC patients at various clinical stages. Discovered alterations in infiltrating immune cells as the illness advanced, along with increased ratios of diminished CD8+ T cells and immunosuppressive M2-like macrophages in advanced stages (98). Chirag Krishna et al. published their work in the year 2021. By conducting scRNA-seq and TCR-seq on 167,283 cells obtained from various tumor tissues, lymph nodes, paracancerous tissues, and peripheral blood of six patients (two without ICB treatment and four with ICB treatment), we discovered variations within and among patients. In ICB-responsive patients, CD8A+ tissue-resident T cells were abundant, while resistant patients exhibited an enrichment of tumor-associated macrophages (TAMs). Furthermore, analysis of TCR trajectories unveiled distinct paths of T cell differentiation in ICB-responsive and non-responsive patients. Several independent cohort studies (99) have correlated the response to ICB and targeted therapy with the transcriptional profiles of tissue-resident T cells and TAMs at the single-cell level. Kevin Bi et al. published their work in the same year. Examined the transcriptional characteristics of individual tumor cells and immune cells in patients with metastatic renal cell carcinoma before and after exposure to ICB. Discovered that cytotoxic T-cell subsets in ICB responders exhibited significant expression of co-inhibitory receptor and ligand molecules. In 2021, Nicholas Borcherding and colleagues utilized single-cell RNA sequencing and T-cell receptor sequencing to examine the diversity in gene expression of 25,688 CD45+ lymphocytes and myeloid cells individually obtained from tumor tissue and blood samples of three ccRCC patients. The analysis of 11,367 immune cells from both the kidney and peripheral blood of four additional ccRCC patients demonstrated a general rise in the populations of CD8+ T cells and macrophages within tumor-infiltrating immune cells, in contrast to normal renal tissues. Furthermore, it identified the MKI67+ proliferative subpopulation as a potential contributor to the progression of ccRCC (100). The latest findings indicate that CD8+ T cells continue to have a significant and intricate impact on the advancement of ccRCC.

#### 4.1.2 Tumor-associated macrophages (TAM) in bone marrow-derived cells

Tumor immunity is significantly influenced by myeloid cells, which consist of various cell populations, such as granulocytes and mononuclear phagocytes. Macrophages, which are phagocytic cells, form a diverse group with intricate characteristics in the tumor microenvironment TME. Macrophages can eradicate cancerous cells by either triggering programmed cell death in tumor cells through engulfment or by releasing substances that can cause their demise. Macrophages not only have the ability to directly kill tumors, but they also have a crucial function in controlling tumor advancement by employing processes like angiogenesis, fibrosis, and immune surveillance. The research on ccRCC also emphasizes Tumor-associated macrophage TAM, where the polarization of TAM towards M2-like macrophages and macrophages with anti-inflammatory characteristics is a prevalent occurrence in progressive ccRCC. Chirag Krishna et al. have reported that certain TAM phenotypes can decrease the immune infiltration temperature of ccRCC. It was determined that TAM phenotypes with elevated HLA expression enhance resistance to Immune Checkpoint Inhibitors (ICIs), as examined through single-cell sequencing techniques (99). In their study, Aleksandar Obradovic and colleagues discovered a distinct group of macrophages called tumor-specific macrophages (TAMs). These TAMs were identified by analyzing the expression of TREM2/APOE/C1Q using single-cell RNA sequencing (scRNA-seq) on different cell subpopulations obtained from surgically removed tumors and nearby tissues of untreated ccRCC patients. The findings were further confirmed using spatially resolved, quantitative multispectral immunofluorescence. The infiltration was of macrophages positive for TREM2/APOE/c1q was found to be a possible biomarker for predicting the recurrence of ccRCC and a potential target for therapy (101). The immunosuppressive tumor microenvironment in ccRCC may be regulated by TAM through complement activation and metabolic reprogramming, as observed in previous reports (98, 99) utilizing single-cell sequencing.

#### 4.1.3 B cell

B lymphocytes are components of the humoral immune system that contribute to humoral immunity. They react to infected or cancerous cells and transform into memory B cells or plasma cells. The plasma cells produce immunoglobulin IGs (antibodies) to attach to and counteract antigens. Historically, B lymphocytes lack anti-cancer properties, yet there is speculation regarding their potential contribution to the development of the tumor immune microenvironment in ccRCC (102, 103). After extensively reviewing numerous literature sources, we were unable to locate any studies or analyses about single-cell sequencing of B-cell-related populations in ccRCC. Consequently, we have decided not to delve into this topic within the scope of this paper.

#### 4.1.4 NK cell

Natural killer (NK) cells are usually lymphoid cells with innate-like characteristics that carry out cytotoxic activities independently of MHC specificity. As a result, they can enhance the elimination of tumors restricted by MHC through the actions of cytotoxic T cells. NK cells in ccRCC have demonstrated dual functions of inhibiting tumor growth and promoting tumor advancement. The secretion of cytokines and growth factors by nK cells can facilitate the growth of ccRCC tumors and promote angiogenesis. Additionally, they can suppress the function of other immune cells that might target cancerous growths. Nevertheless, natural killer cells can additionally impede ccRCC by directly identifying and eliminating malignant cells via various pathways, such as the secretion of toxic particles and attachment to receptors that induce cell death. Several factors, such as the disease stage, the existence of different NK cell subsets, and the composition of TME (104), could influence the equilibrium between the pro- and anti-tumorigenic functions of NK cells in ccRCC. Nevertheless, similar to B cells, we were unable to find any pertinent research examining NK cells through the utilization of single-cell methodologies.

## 5 Development of immunotherapy for ccRCC

In the last twenty years, there has been an increasing exploration of immunotherapeutic targets, accompanied by the ongoing improvement of the ccRCC tumor microenvironment concept. At first, immunotherapy was employed to interfere with the advancement of tumors by utilizing leukocyte-lending cytokines and interferon, however, the outcomes were unsatisfactory and accompanied by numerous complications (105, 106). The development of tyrosine kinase inhibitors, also referred to as vascular endothelial growth factor receptor (VEGF) inhibitors, under the anti-angiogenesis theory significantly enhanced the survival rates of patients, both in terms of progression-free survival (PFS) and overall survival (OS) (107). Nevertheless, the challenges posed by the resistance and adverse reactions of tyrosine kinase inhibitors have significantly hindered clinical treatment. The emergence of immune checkpoint inhibitors (ICIs) has offered a fresh approach to treating metastatic ccRCC, gradually becoming a viable alternative to anti-angiogenic medications. Monoclonal antibodies known as immune checkpoint inhibitors (ICIs) activate anti-tumor immune responses by blocking inhibitory molecules on T-cells or their ligands on tumor cells. PD-1 and CTLA-4, which have demonstrated effectiveness, are the ICIs that have been extensively researched in ccRCC treatment. PD-I is a receptor that inhibits T-cell activation and promotes immune evasion by binding to PD-Ll, a ligand expressed in tumor cells and other immune cells (108). Navumab, pembrolizumab, and atezolizumab are among the PD-1/PD-L1 inhibitors that have received approval for treating advanced ccRCC. However, ICI resistance mechanisms can be primary or innate, secondary or acquired (109). The factors comprise loss of neoantigens, impaired presentation of antigens, alternative checkpoints of the immune system, and impaired signaling of interferons. The main mechanisms for resistance development include the increase in programmed death ligand 1 (PD-L1) expression due to interferon-γ and the induction of immunosuppressive molecule expression (110). So new immune checkpoints are being proposed, e.g., TIM-3, LAG-3, TIGIT, etc. (111) New therapeutic approaches try to overcome these resistance mechanisms, but their effectiveness and feasibility need to be evaluated by further experiments (112).

## Data availability statement

The data used to support the findings of this study are available from the corresponding author upon request.

## Author contributions

GW: Conceptualization, Formal analysis, Funding acquisition, Writing – original draft. YL: Data curation, Investigation, Methodology, Writing – review & editing.

## Glossary

**Table:**

ccRCC	Clear cell renal cell carcinoma
ECM	Extracellular matrix
scRNA-seq	Single-cell RNA sequencing
scProteomics-seq	Single-cell proteome sequencing
scDNA-seq	Single-cell DNA sequencing
ICB	Immune checkpoint blockade
TCR	T cell receptor
TAMs	Tumor associated macrophages
RCC	Renal cell carcinoma
PRCC	Papillary renal cell carcinoma
chRCC	Chromophobe renal cell carcinoma
UV	Ultraviolet
VHL	Von Hippel Lindau
NGS	Next-Generation Sequencing
scWGS	Single cell whole genome sequencing
IVT	*In vitro* transcription
dNTPs	Deoxyribonucleotide triphosphates
MMLV	Moloney Murine Leukemia Virus
TSO	Template-switching oligo
MMLVRT	Moloney murine leukemia virus reverse transcriptase
WTA	Whole transcriptome amplification
UMI	Utilizes unique molecular identifiers
FACS	Fluorescence activated Cell Sorting
RT	Reverse transcription
PDMS	Polydimethylsiloxane
MALBAC	Multiple annealing and looping-based amplification cycles
GEM	Gel in Emulsion
TME	Tumor microenvironment
ECM	Extracellular matrix
TIME	Tumor immune microenvironment
MHC	Histocompatibility complex molecules
PD-1	Protein 1
CTLA-4	Cytotoxic T-lymphocyte-associated protein 4
MDSCs	Myeloid-derived suppressor cells
TIL	Tumor-infiltrating lymphocytes
NK	Natural killer
VEGF	Vascular endothelial growth factor receptor
PFS	Progression-free survival
OS	Overall survival
